# Microbial load and antibiotic resistance in raw beef preparations from northwest Spain

**DOI:** 10.1002/fsn3.1319

**Published:** 2019-12-26

**Authors:** María González‐Gutiérrez, Camino García‐Fernández, Carlos Alonso‐Calleja, Rosa Capita

**Affiliations:** ^1^ Department of Food Hygiene and Technology, Veterinary Faculty University of León León Spain; ^2^ Institute of Food Science and Technology University of León León Spain

**Keywords:** beef preparations, *Escherichia coli*, microbiological quality, resistance to antibiotics, Spain

## Abstract

Beef preparations (meatballs, minced meat, hamburgers, white sausages, and red sausages) from northwest Spain were tested. Microbial counts ranged from 0.70 ± 0.00 log_10_ cfu/g (enterococci) to 9.57 ± 0.37 log_10_ cfu/g (psychrotrophs). In 73.3% of cases, total aerobic counts were higher than the microbiological limits set for the end of the manufacturing process in the European Union (EU Regulation 2073/2005). Forty *Escherichia coli* isolates were tested against thirteen clinically important antibiotics (disk diffusion method; CLSI). Three of the strains (7.5%) were susceptible to all the antibiotics, four (10.0%) showed resistance or reduced susceptibility to one antibiotic, and 33 (82.5%) were multiresistant (with resistance or reduced susceptibility to between two and eight antibiotics), with an average of 1.85 resistances per strain. The highest rates of resistance were observed for two antimicrobials widely used on cattle farms (cefalotin and tetracycline). The findings in this research emphasize the need to correctly handle beef preparations with the aim of reducing risks to consumers.

## Graphical abstract

Raw beef preparations show high microbial loads and are a reservoir of multi resistant bacteria. The need to correctly handle beef preparations and the crucial need to reduce resistance to antibiotics in the bacteria present in these foodstuffs are both underlined.

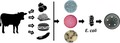

## 1 INTRODUCTION

Beef production is the third largest meat industry worldwide, producing some 65 million tonnes in total, exceeded only by pig and poultry farming (Cameron & McAllister, 2016). In 2013, 9.32 kg of beef were consumed per head of world population, with per capita per year figures for pig meat and poultry standing at 16.02 and 14.99 kg, respectively. Beef consumption is high in the European Union, at 14.89 kg per person per year (FAOSTAT, 2019). A certain percentage of beef is consumed in the form of meat preparations. Regulation (EC) 853/2004 defines meat preparations as fresh meat, including meat that has been reduced to fragments, which has had foodstuffs, seasonings or additives added to it or has undergone processes insufficient to modify the internal muscle fiber structure of the meat and thus eliminates the characteristics of fresh meat (European Parliament and Council, 2004).

The extensive consumption of beef leads to concerns that the products marketed should be safe, have a low spoilage rate, and the stipulated composition, packaging, color, taste, and appearance. In such a scenario, products excessively contaminated with microorganisms are undesirable (Del Río, Panizo‐Morán, Prieto, Alonso‐Calleja, & Capita, 2007). There are several microbial parameters (total aerobic counts ‐ TACs‐ psychrotrophic microorganisms, enterobacteria, fecal coliforms, and enterococci) that are interesting to evaluate for meat because they act as indicators of its microbiological quality and the level of hygiene in the processes of production, handling, and maintaining an unbroken cold chain, also helping to predict the potential shelf life of products (Alonso‐Calleja, Martínez‐Fernández, Prieto, & Capita, 2004; Álvarez‐Astorga, Capita, Alonso‐Calleja, Moreno, & García‐Fernández, 2002).

Bacterial resistance to antibiotics has grown at an alarming rate over the last few years and has been described as one of the greatest threats to public health, and consequently one of the main challenges for humanity in the twenty‐first century. It has been considered as one of the biggest problems for health systems in Europe, as a worldwide pandemic, and even as a potential global health catastrophe (Capita & Alonso‐Calleja, 2013).

In 2015, the European Union (EU) and the European Economic Area (EEA) saw more than 33,000 deaths attributable to infections caused by bacteria resistant to antibiotics, implying a loss of 874,541 disability‐adjusted life‐years (DALYs; Cassini et al., 2019). Infections by resistant bacteria lead to 23,000 deaths annually in the United States (Cecchini, Langer, & Slawomirski, 2015). Moreover, these figures are on a rising trend, with estimates that in three decades’ time ten million deaths will occur each year worldwide as a result of antibiotic‐resistant bacteria, a higher figure than the 8.2 million deaths that will be caused by cancer. These figures should be contrasted with the 700,000 deaths attributable to antibiotic resistance in 2014 (O’Neill, 2016). The financial consequences of resistance to antibiotics are also of considerable weight, with estimates that every year these infections cost the healthcare systems of EU and EEA countries 1.1 thousand million euros (OECD, 2019).

The presence of antibiotic‐resistant bacteria in foods is a direct risk for consumers due to the potential of these microorganisms to cause hard‐to‐treat foodborne infections. There is also an indirect risk of horizontal transfer of resistance genes to pathogenic microorganisms, including among unrelated genera, at various points along the food chain such as in the animals reared for consumption, in foodstuffs, on surfaces and equipment in the food‐processing industry, and so on (Capita & Alonso‐Calleja, 2013). *Escherichia coli* has a striking ability to acquire antibiotic resistance genes as a result of the efficient horizontal transfer mechanisms these microorganisms have developed over time. Hence, strains of this bacterial group act as reservoirs of resistance genes, which is a worrying fact in the context of public health since there is a high likelihood of gene transfer to other, pathogenic, bacteria. Moreover, this circumstance allows this bacterial group to be used as sentinel for resistance to antibiotics (SCENIHR, 2009).

Monitoring resistance to antibiotics is essential not only to obtain information about the magnitude of this problem and trends within it, but also to plan and monitor the effectiveness of any control measures introduced. Monitoring antimicrobial resistance in indicator *E. coli* from food‐producing animals and food products of animal origin has been mandatory under EU legislation since 2014 (EFSA & ECDC, 2019). However, there is very limited information about resistance to antibiotics in strains of *E. coli* from beef and beef products, especially in northwest Spain.

The aims of the present work were to gain awareness of the microbiological quality of beef preparations purchased in three different retail establishments in northwest Spain, to compare levels of contamination in various types of beef preparations and to determine patterns of antibiotic resistance in strains of *E. coli* isolated from these foodstuffs.

## 2 MATERIAL AND METHODS

### 2.1 Meat samples

A total of thirty samples of beef preparations produced in three retail establishments in the city of León in northwest Spain were analyzed. The establishments involved were two butcher's shops (A and B) and a supermarket (C). Samples from meatballs, minced meat, hamburgers, white sausages, and red sausages were taken on site, transported immediately to the laboratory, and kept under refrigeration (4 ± 1°C) for a maximum of four hours prior to the start of the analyses. Six samples of each type of product were investigated.

### 2.2 Microbiological analysis

Using sterile tweezers and a scalpel, 25 g of product were taken from each sample and placed in a homogenization bag together with 225 ml of sterile 0.1% peptone water (Oxoid Ltd.). These samples were homogenized (Masticator, IUL Instruments) for two minutes. Subsequent decimal dilutions were performed in test tubes with 9 ml of the same diluent. Table 1 shows the culture media, incubation conditions, and references used for each of the microbial groups studied. All inoculations were carried out in duplicate. Plates with between 25 and 250 colonies (spread‐plate technique) or between 30 and 300 colonies (pour‐plate technique) were counted, and mean counts were calculated. All culture media were purchased from Oxoid.

**Table 1:** Culture media, incubation times, temperatures, and references used for microbiological analysis

Microbial group	Culture medium	Incubation		Reference
		T (°C)	Time	
Aerobic plate count (APC)^a^	Plate count agar (PCA)	30	72 hr	Jay (2002)
Psychrotrophs^a^	Plate count agar (PCA)	7	10 d	Cousin, Jay, & Vasavada (2001)
*Enterobacteriaceae* b, c	Violet red bile glucosa agar (VRBGA)	35	24 hr	Baird, Corry, & Curtis (1987)
Fecal coliforms^b^, ^c^	Violet red bile agar (VRBA)	44	24 hr	Baird et al. (1987)
Enterococci^b^	Kanamycin aesculin azide agar (KEA)	42	24 hr	Baird et al. (1987)

a Spread‐plate technique (0.1 ml).

b Pour‐plate technique (1 ml).

c Overlay procedure.

### 2.3 Isolation and identification of *Escherichia coli* isolates

Between four and six colonies were taken from each violet red bile agar (VRBA) plate for later identification. The strains were streaked onto plates of tryptone soy agar (TSA) and then incubated for 24 hr at 44 ± 1°C to obtain pure cultures. The resultant pure cultures were examined for colony and cell morphology, Gram stain, and oxidase and catalase activities. Presumptive *E. coli* strains were confirmed using a miniaturized *E. coli* test system (Liofilchem s.r.l., Teramo, Italy) in accordance with the manufacturer's instructions. A total of forty *E. coli* isolates (eight strains from each type of product) were selected for later antibiotyping. The strains were stored at −50°C in tryptone soy broth (TSB) with 20% glycerol.

### 2.4 Antibiotic resistance study

The susceptibility of the strains was tested against a panel of thirteen clinically important antimicrobials. The following antibiotic disks (Oxoid) were used to perform antibiograms by means of the disk diffusion method on Mueller‐Hinton agar: gentamicin (CN; 10 µg), penicillin G (P; 10 units), cefazolin (KZ; 30 µg), cefoxitin (FOX; 30 µg), cefotaxime (CTX; 30 µg), cefalotin (KF; 30 µg), cefepime (FEP; 30 µg), chloramphenicol (C; 30 µg), nalidixic acid (NA; 30 µg), ciprofloxacin (CIP; 5 µg), tetracycline (TE; 30 µg), ampicillin‐sulbactam (SAM; 20 µg), and amoxicillin–clavulanic acid (AMC; 30 µg). After incubation at 37°C for 18 to 24 hr, inhibition zones were measured and scored as susceptible, intermediate (reduced susceptibility), or resistant according to the Clinical and Laboratory Standards Institute (CLSI, 2019) guidelines. *Escherichia coli* ATCC 25922 and *Staphylococcus aureus* ATCC 29213 were used as reference strains for antibiotic disk control.

### 2.5 Statistical analysis

The microbial counts were transformed to log_10_ cfu/g. The data were subjected to analysis of variance (ANOVA) techniques using Duncan's multiple range test to separate averages. Significant differences were established for a probability level of 5% (*p* < .05). All the tests were carried out with the Statistica® 8.0 package (Statsoft Ltd.).

## 3 RESULTS

### 3.1 Microbial load in beef preparations

Analysis of variance (ANOVA) was used to investigate the three factors, microbial group (G), establishment (E), and type of beef preparation (T). This highlighted the influence (*p* < .001) of the three factors and their interactions.

Table 2 shows the average counts for all the microbial groups evaluated. Mean values (log_10_ cfu/g) ranged from 0.70 ± 0.00 for enterococci in minced meat and hamburgers from establishment B to 9.57 ± 0.37 for psychrotrophs in meatballs from establishment C. All forty beef samples harbored *E. coli* strains.

**Table 2:** Microbial counts (log_10_ cfu/g) found in various beef‐based preparations from three retail establishments

Microbial group	Establishment	Type of meat preparation				
		Meatballs	Minced meat	Hamburgers	White sausages	Red sausages
Total aerobic counts (TACs)	A	7.92 ± 1.12^a^_ab_	7.78 ± 1.27^a^_a_	7.68 ± 0.97^a^_a_	7.18 ± 1.20^a^_a_	6.80 ± 1.16^a^_a_
	B	7.78 ± 0.79^a^_a_	6.92 ± 1.12^ab^_a_	5.66 ± 2.02^b^_b_	6.91 ± 0.82^ab^_a_	5.88 ± 2.22^b^_ab_
	C	8.91 ± 0.98^a^_b_	7.77 ± 0.75^a^_a_	8.19 ± 0.82^a^_a_	5.24 ± 2.14^b^_b_	4.56 ± 1.96^b^_b_
Psychrotrophs	A	8.01 ± 1.37^a^_a_	7.86 ± 0.83^a^_a_	8.04 ± 0.89^a^_a_	7.70 ± 0.81^a^_a_	7.33 ± 0.78^a^_a_
	B	8.27 ± 0.47^a^_a_	5.46 ± 1.89^b^_b_	5.66 ± 2.16^b^_b_	7.47 ± 0.90^ac^_a_	6.21 ± 1.74^bc^_a_
	C	9.57 ± 0.37^a^_b_	7.48 ± 1.12^b^_a_	7.89 ± 1.32^ab^_a_	4.21 ± 2.71^c^_b_	3.68 ± 1.75^c^_b_
Enterobacteria	A	3.10 ± 2.02^a^_a_	1.02 ± 0.75^b^_a_	2.40 ± 1.51^a^_a_	2.04 ± 1.21^ab^_a_	2.77 ± 1.46^a^_a_
	B	1.43 ± 1.10^a^_b_	0.83 ± 0.30^b^_a_	0.72 ± 0.09^b^_b_	0.72 ± 0.09^b^_b_	0.93 ± 0.44^b^_b_
	C	3.63 ± 1.93^a^_a_	2.93 ± 1.82^ab^_b_	1.63 ± 1.26^bc^_ab_	2.06 ± 1.89^bc^_a_	1.28 ± 0.86^c^_b_
Fecal coliforms	A	3.02 ± 1.82^a^_a_	1.65 ± 1.07^b^_a_	1.97 ± 1.49^ab^_a_	2.39 ± 1.41^ab^_a_	1.64 ± 1.45^b^_a_
	B	1.22 ± 1.25^a^_b_	0.75 ± 0.12^ab^_b_	0.70 ± 0.00^b^_b_	0.75 ± 0.17^ab^_b_	0.75 ± 0.12^b^_b_
	C	1.35 ± 1.04^a^_b_	1.08 ± 0.84^a^_ab_	0.95 ± 0.59^a^_b_	1.03 ± 0.80^a^_b_	0.75 ± 0.12^a^_b_
Enterococci	A	0.90 ± 0.32^a^_a_	1.19 ± 0.76^a^_a_	1.80 ± 1.46^ab^_a_	2.49 ± 1.64^b^_a_	3.97 ± 1.29^c^_a_
	B	0.72 ± 0.09^a^_a_	0.70 ± 0.00^a^_b_	0.70 ± 0.00^a^_b_	0.72 ± 0.09^a^_b_	0.95 ± 0.42^b^_b_
	C	0.82 ± 0.27^a^_a_	0.72 ± 0.09^a^_b_	1.16 ± 1.23^a^_ab_	0.98 ± 0.46^a^_b_	0.95 ± 0.46^a^_b_

Note

Averages in the same row sharing one or more superscript letters show no significant differences between them (*p* ≥ .05). Averages in the same column for the same microbial group sharing one or more subscript letters show no significant differences between them (*p* ≥ .05).

**Figure 1:**
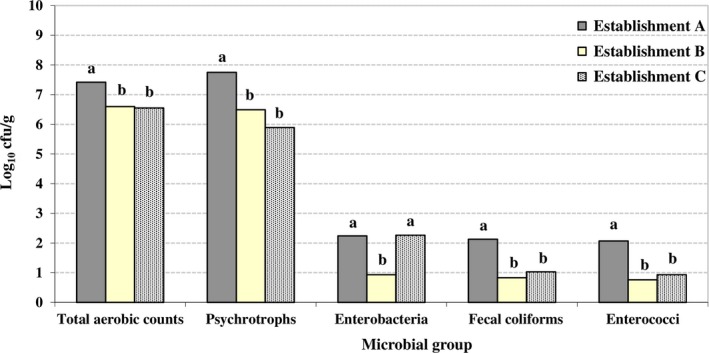
Microbial counts (log_10_ cfu/g) from beef‐based preparations (combined values for the different types of products examined) from three establishments (A, B and C). Values for the same microbial group sharing one or more letters show no significant differences between them (*p* ≥ .05)

**Figure 2:**
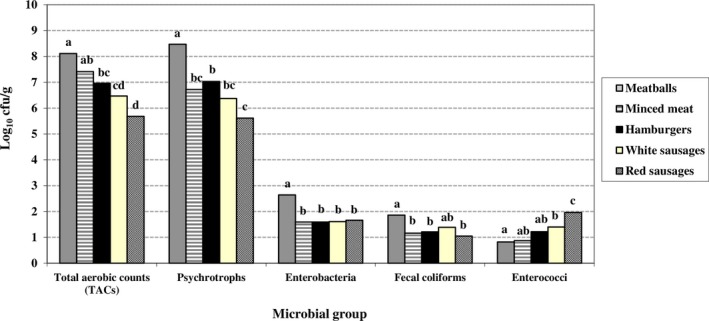
Microbial counts (log_10_ cfu/g) from the various beef‐based preparations analyzed (combined values for the various establishments: A, B and C). Values for the same microbial group sharing one or more letters show no significant differences between them (*p* ≥ .05)

The levels of each microbial group by establishment and type of product are shown in Figures 1 and 2, respectively. The highest counts were recorded in establishment A for TACs, psychrotrophs, fecal coliforms, and enterococci, and in establishments A and C for enterobacteria. The lowest microbial levels were observed in establishment B. Similarly, the kind of meat preparation impacted on results, with red sausages having the lowest counts for all microbial groups except for enterococci (Figure 2).

### 3.2 Antibiotic resistance in *E. coli* isolates from beef preparations

Only three *E. coli* strains (7.5%) were susceptible to all the antibiotics, four (10.0%) were resistant or intermediate to one antibiotic, and thirty‐three (82.5%) were multiresistant (resistant or intermediate to two or more antibiotics). Of these latter strains, six (15%) showed resistance or reduced susceptibility to two antibiotics, eight (20%) to three antibiotics, five (12.5%) to four antibiotics, seven (17.5%) to five antibiotics, two (5%) to six antibiotics, three (7.5%) to seven antibiotics, and two (5%) to eight antibiotics. The average number of resistances per strain was 1.85. Grouping together the strains with resistance and those with reduced susceptibility, the number of resistances per strain was 3.60.

**Figure 3:**
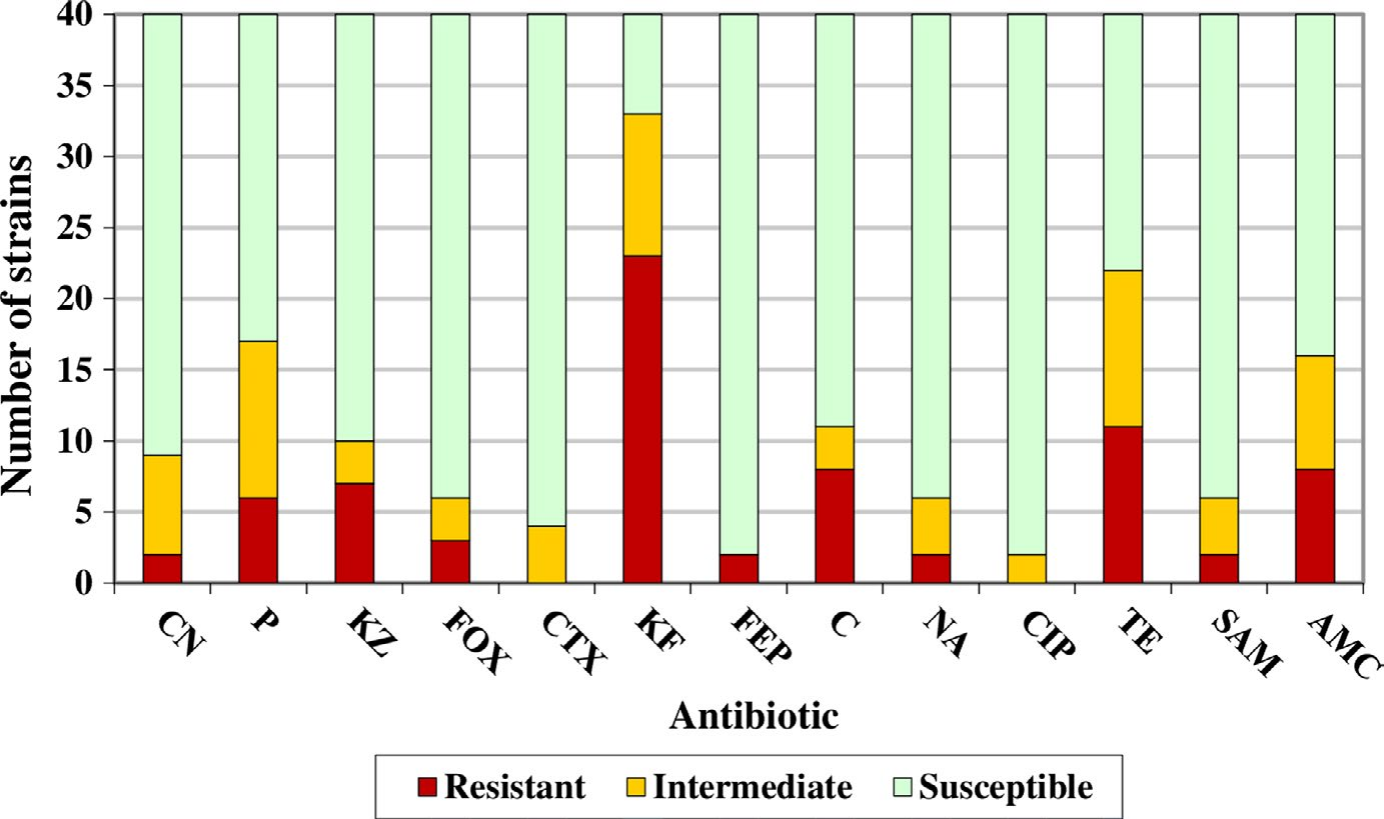
Number of strains resistant, intermediate (having reduced susceptibility), or susceptible to each of the antibiotics examined. AMC, amoxicillin‐clavulanic acid; C, chloramphenicol; CIP, ciprofloxacin; CN, gentamicin; CT X, cefotaxime; FEP, cefepime; FOX, cefoxitin; KF, cefalotin; KZ, cefazolin; NA, nalidixic acid; P, penicillin G; SAM, ampicillin‐sulbactam; TE, tetracycline

Figure 3 shows the number of strains resistant to each of the antibiotics examined. Noteworthy among the results is the high percentage of isolates with resistance or reduced susceptibility to cefalotin (33 strains, 82.5% of isolates) and tetracycline (22 strains, 55.0% of isolates).

## 4 DISCUSSION

### 4.1 Microbial loads in beef preparations

Total aerobic count (TAC) has been used as a criterion to predict the shelf life of meat, given that the presence of this microbial group in large numbers may cause rapid alterations in the product. Counts between 10^7^ and 10^8^ cfu/g are generally associated with the beginning of changes in organoleptic properties such as appearance, smell, and taste (Nychas, Skandamis, Tassou, & Koutsoumanis, 2008). TACs can also be indicators of inappropriate processing, so determining their presence is a way of monitoring good manufacturing practices (GMP). While high levels of these microorganisms do not necessarily imply potential risks for human health, the importance of TAC lies in the fact that it is an expression of the hygienic quality of foodstuffs (Del Río et al., 2007).

The microbiological limit for TAC in meat preparations set by European Union standards is 6.7 log units/g (Commission of the European Communities, 2005). Guidelines and recommendations have been developed in addition to these legal requirements to monitor the microbial quality of meat preparations. According to GMP guidelines, the level of total microbiological contamination for raw meat preparations should not exceed 5 (maximum 7) log units/g (ICMSF, 2011; IFST, 1997). According to the microbiological guidelines laid down by the Laboratory of Food Microbiology and Food Preservation of the University of Ghent (LFMFP‐UGent), the maximum admissible figure for TAC is 6.5 log units/g (Uyttendaele, Jacxsens, De Loy‐Hendrickx, Devlieghere, & Debevere, 2010). Of the average values for TAC in the present study, 73.3% exceeded 6.7 log units (Table 2), with lower figures found only in hamburgers and sausages (especially red sausages). Only red sausages from establishment C had average values below 5 log units. Nevertheless, it must be pointed out that the microbiological criteria indicated above refer to the end of the manufacturing process, while the samples tested in this research were obtained from retail establishments. A higher level of contamination is to be expected during the shelf life of the product than at the final point of the manufacturing process.

The high TACs recorded in this work coincide with the findings of other researchers that have investigated meat preparations (Andritsos, Mataragas, Mavrou, Stamatiou, & Drosinos, 2012; Tegegne & Ashenafi, 1998; Siriken, 2004). These high microbial levels may be due to the heavy contamination of the raw materials or to inappropriate processing conditions such as excessively high temperatures that favor the contamination of meat preparations and the later proliferation of microorganisms. To this effect, it is often suggested that microorganisms can enter meat preparations not only from the meat, spices, and other ingredients, but also from the processing environment, equipment, and handlers, all of which are factors that can have a significant impact on the microbiological status of the end products. These results emphasize the need to encourage consumers to cook minced beef thoroughly and to adhere to safe food handling guidelines. GMPs for the slaughtering and processing of ground beef should also be acknowledged as strategies to control microbial load.

Counts of psychrotrophs are particularly relevant for products that are kept refrigerated, a storage condition in which these microorganisms can multiply. Of the samples taken, 26.7% presented values exceeding 8 log cfu/g. However, none of the samples showed organoleptic signs of spoilage. These findings do not coincide with the results of Dainty and Mackey (1992) or those of Griffiths, Phillips, and Muir (1981), who indicate that levels of 6 to 8 log_10_ cfu/g of microorganisms are sufficient to produce off‐odors and appearance defects in meat. Stanbridge and Davies (1998) also state that levels of psychrotrophs over 7 to 8 log units trigger strange smells and surface sliminess in meat. Contrarily, the results presented here are in agreement with the findings of El‐Leithy and Rashad (1989), who report that none of the samples of minced meat and mincemeat products analyzed as having more than 8 log_10_ cfu/g had off‐odors. Goepfert (1976) also found that aerobic plate counts equal to or greater than 7.7 log_10_ cfu/g did not produce any organoleptic alteration in meat.

The samples investigated showed similar figures for TACs (incubated at 30°C) and psychrotrophic microorganisms (incubated at 7°C). These findings do not coincide with those in an earlier work (Álvarez‐Astorga et al., 2002), where higher levels of psychrotrophic microorganisms than TACs were recorded in meat and meat preparations stored under refrigeration. In fact, psychrotrophs are the microorganisms of choice for assessing the microbiological quality of refrigerated meat, and the fact that similar levels of these two microbial groups were observed in the present work suggests that storage temperatures were not sufficiently low.

Most of the enterobacteria and fecal coliforms found in meat come from contamination with feces, so their presence in large numbers may indicate poor hygiene in the slaughterhouse from which the meat is sourced, insufficiently hygienic handling, inappropriate storage, or a combination of these (Andritsos et al., 2012). It should be noted that all the counts for these microbial groups were within the guideline microbiological criteria, which states that this should not exceed 2 (maximum 4) log units/g (ICMSF, 2011; IFST, 1997) or 3.5 (maximum 4.5) log units/g (Uyttendaele et al., 2010). Other authors have noted similar counts to those in the present study regarding the presence of enterobacteria and fecal coliforms in beef (Lunning et al., 2011). The detection of *E. coli* strains in all the meat analyzed is also a frequent finding for red meat and poultry samples (Álvarez‐Fernández, Cancelo, Díez‐Vega, Capita, & Alonso‐Calleja, 2013).

The presence of a large number of enterococci in foodstuffs implies inadequate hygiene practices or exposure of the food to conditions that allow undesirable bacteria to excessively multiply (Franz, Holzapfel, & Stiles, 1999). These microorganisms can play an important role as indicators of deficiencies in cleaning and disinfection practices, given their considerable resistance not only to drying out, but also to both high and low temperatures and to detergents and disinfectants. This strong resistance also explains why this microbial group is not a valid indicator of fecal contamination (Thian & Hartman, 1981). The levels of enterococci recorded in this study are similar to those previously observed in prepared poultry products (Buzón‐Durán, Capita, & Alonso‐Calleja, 2017).

When the data were grouped by establishment, the highest microbial counts were found in establishment A, followed by establishment C. Establishment B’s results were the most satisfactory. Other authors have also found differences between types of establishment. To this effect, Andritsos et al. (2012) compared the microbiological quality in specialist butcher's shops and the meat sections of supermarkets. They reported deficient outcomes in all the supermarkets and marginally acceptable results in the butcher's shops.

In the present work, establishment A was a small butcher's shop with staff with many years’ experience, but little specific training in food hygiene and little engagement with the Hazard Analysis and Critical Control Points (HACCP) system in force. Furthermore, there were no set microbiological specifications with the slaughterhouse supplying the meat. These may be explanatory factors as to why high microbial counts were observed in this establishment. Establishment B was a medium‐sized butcher's specializing in the sale of high‐quality products, whose personnel had less experience but had undergone more specific training in food hygiene, and was highly committed to implanting the HACCP system. Moreover, the slaughterhouse supplying establishment B with meat was small‐scale and belonged to a meat brand of recognized prestige where hygiene quality is a highly controlled aspect. Establishment C was the meat section of a supermarket. In this kind of establishment, all staff are trained in food hygiene by the quality control department of the business prior to taking up employment, and considerable resources are allocated to implanting the HACCP system. Furthermore, the chain of supermarkets to which this establishment belongs had agreed upon some microbiological specifications with its supplier and any failure to comply with them would result in rejecting the meat.

Differences in microbiological quality were observed among the various kinds of meat preparations. Noteworthy among the results were the low counts for most of the microbial groups in red (spiced) sausages, the exception being enterococci. This finding points to the possibility that the spices and other additives used in the production process, for instance paprika, may have a bactericidal and/or bacteriostatic effect on certain microorganisms, as has been suggested by other researchers (Gottardi, Bukvicki, Prasad, & Tyagi, 2016).

### 4.2 Antibiotic resistance in *E. coli* isolates from beef preparations

The forty strains of *E. coli* isolated were tested against a panel of thirteen clinically important antimicrobials. An alarming 92.5% of the strains were resistant or intermediate to one or more antibiotics, with over 80% of the strains presenting multiresistance (with resistance or reduced susceptibility to two or more antibiotics). The presence of bacteria resistant to antibiotics in red meat and poultry is a frequent finding (Álvarez‐Fernández, Alonso‐Calleja, García‐Fernández, & Capita, 2012; Buzón‐Durán et al., 2017; Capita, Álvarez‐Fernández, Fernández‐Buelta, Manteca, & Alonso‐Calleja, 2013; Davis et al., 2018; Hussain et al., 2017; Koo & Woo, 2011; Ojer‐Usoz et al., 2013). Some authors have even observed percentages of resistant strains of up to 100% (Carramiñana, Rota, Agustín, & Herrera, 2004). Nonetheless, the percentage of multiresistant strains noted in this investigation is high, and much greater than the average value of 27.7% found across the European Union for strains of *E. coli* of bovine origin (EFSA & ECDC, 2019). The average number of resistances per strain observed in the present study, at 1.85, was lower than the figures recorded by Logue, Sherwood, Olah, Elijah, and Dockter (2003) in the United States. These researchers found that strains of enterobacteria originating in poultry meat had resistance to an average of 4.0 antimicrobials.

Bacteria resistant to antibiotics can cause infections in consumers through the consumption of foodstuffs in instances of cross‐contamination or when food is insufficiently cooked. Given that clinical treatment for infections is rendered complex when strains are resistant to antibiotics, the presence of multiresistant bacteria in food is a cause for great concern (Capita & Alonso‐Calleja, 2013).

Notably, at least 10% of strains showed resistance or reduced susceptibility to penicillin G, cefazolin, cefalotin, chloramphenicol, tetracycline, or amoxicillin–clavulanic acid, which are classified as “critically important antimicrobials” (amoxicillin–clavulanic acid) or “highly important antimicrobials” (penicillin G, cefazolin, cefalotin, chloramphenicol, and tetracycline) for human medicine (WHO, 2019). According to the World Organization for Animal Health (OIE, 2018), penicillin G, tetracycline, and amoxicillin–clavulanic acid are “critically important antimicrobial agents,” while cefazolin and cefalotin are categorized as “highly important antimicrobial agents” in veterinary medicine (OIE, 2018). High levels of resistance to such antimicrobials have also been reported by other authors in bacteria isolated from beef (Cameron & McAllister, 2016; EFSA & ECDC, 2019; Hiroi et al., 2012; Jaja, Bhembe, Green, Oguttu, & Muchenje, 2019; Messele et al., 2017).

The large number of resistant strains in foods reported in most of the publications consulted would appear to be related to the use of antibiotics in various contexts (agriculture, animal production, and clinical practice), which has had an enormous impact on microbial populations and encouraged the selection of resistant bacteria (Buzón‐Durán, Capita, & Alonso‐Calleja, 2018). To this effect, selective pressure has been exerted by the use of antibiotics, particularly when they are used incorrectly, for instance at a subinhibitory doses in animal production and human medicine, which has been identified as the main cause of resistance to antibiotics that has emerged over recent decades (Alonso‐Hernando, Prieto, García‐Fernández, Alonso‐Calleja, & Capita, 2012; Álvarez‐Fernández et al., 2012). On this point, the World Health Organization has noted that the use of antibiotics in animal production has a marked impact on the prevalence of resistance to antibiotics in human infections, publishing a range of documents dealing with this issue (WHO, 2009). To this effect, in the present study considerable prevalence of resistance to cefalotin and tetracycline, antibiotics widely used in animal production, was observed (Cameron & McAllister, 2016; De Briyne, Atkinson, Pokludová, & Borriello, 2014).

The high prevalence of resistance or reduced susceptibility to antibiotics noted in the present work is, nonetheless, surprising given that some of the substances to which the strains showed resistance are not employed in veterinary medicine in Spain. The toxicological effects on consumers (carcinogenicity and mutagenicity) of chloramphenicol, for example, led to the prohibition of the use of this antibiotic in animal production in the European Union almost thirty years ago. This substance is included in Annex IV to Council Regulation 2377/90, which lays down zero tolerance for chloramphenicol in all foods of animal origin. Notably, although this substance has not been used on cattle farms in Spain for many years now, coresistance or cross‐resistance mechanisms might be at play in the resistance to chloramphenicol observed, a fact noted by various authors (Capita & Alonso‐Calleja, 2013; van Duijkeren, Wannet, Houwers, & van Pelt, 2003; Yildirim, Gonulalan, Pamuk, & Ertas, 2011). Multiple resistance to antibiotics has recently been associated with plasmids of large size, which are transferable between strains. These transferable plasmids carry mobile genetic DNA elements (integrons) that often contain numerous genes for resistance to antibiotics, which are transferred simultaneously to other bacteria, where they are jointly expressed (Schroeder, Hoog, & Helmuth, 2004). According to Martins da Costa, Oliveira, Ramos, and Bernardo (2011), the phenomenon of coselection is hugely important in the persistence of multiresistant strains which are, moreover, stable and capable of lingering on farms long after any selective pressures have disappeared (Song et al., 2008).

Along these lines, some years ago it was demonstrated that the use of antibiotics modifies the resistance genes present in bacterial communities (the resistome). The effects on this set of genes persist for years even in the absence of any contact with antibiotics (Agga et al., 2019; Johnsen et al., 2011; Sommer & Dantas, 2011). Smith et al. (2007) found that successive exposures to antibiotics create a resistance that is stable over time and that resistant strains can compete with susceptible strains even when there is no selective pressure. Hence, rates of resistance to antibiotics on a farm need not be directly linked to their use in this environment (Luangtongkum et al., 2006).

## 5 CONCLUSIONS

The samples of beef preparations studied presented high levels of microorganisms. In 73.3% of cases, total aerobic counts were higher than the limits set in microbiological criteria for the end of the manufacturing process. Striking differences in the microbial counts obtained were seen depending on the establishment where the products were purchased and on the type of meat preparation involved. The results from the present study provide evidence that strains of *E. coli* in beef preparations pose a major potential risk (both direct and indirect) to consumers, given the considerable rates of resistance or reduced susceptibility to antibiotics that were found. This is a worrying fact from the viewpoint of public health, pointing to a need to take measures to reduce the rates of resistance to antibiotics in the bacteria present in these foodstuffs.

## CONFLICT OF INTEREST

The authors declare that there is no conflict of interest.

## ETHICAL APPROVAL

This study does not involve any human or animal testing.
